# How primary care reforms influenced health indicators in Manisa district in Turkey: Lessons for general practitioners

**DOI:** 10.1080/13814788.2017.1410538

**Published:** 2017-12-15

**Authors:** Celalettin Cevik, Kaan Sozmen, Bulent Kilic

**Affiliations:** ^a^ Department of Nursing, School of Health, Balikesir University Balikesir Turkey; ^b^ Department of Public Health, Katip Celebi University Medical Faculty Izmir Turkey; ^c^ Department of Public Health, Dokuz Eylul University Medical Faculty Izmir Turkey

**Keywords:** Family physician, health centre, health reform, primary healthcare, general practice

## Abstract

**Background:** Turkish health reforms began in 2003 and brought some significant changes in primary care services. Few studies in Turkey compare the shift from health centres (HC) to family physicians (FP) approach, which was initiated by reforms.

**Objectives:** This study compares health status indicators during the HC period before reforms (2003–2007) and the FP period after reforms (2008–2012) in Turkey.

**Methods:** This study encompasses time series data consisting of the results of a 10-year assessment (2003–2012) in Manisa district. All the data were obtained electronically and by month. The intersection points of the regression curves of these two periods and the beta coefficients were compared using segmented linear regression analysis.

**Results:** The mean number of follow-up per person/year during the HC period in infants (10.5), pregnant women (6.6) and women (1.8) was significantly higher than the mean number of follow-up during the FP period in infants (6.7), pregnant women (5.6) and women (0.9). Rates of BCG and measles vaccinations were significantly higher during the FP period; however, rates of HBV and DPT were same. The mean number of outpatient services per person/year during the FP period (3.3) was significantly higher than HC period (2.8). Within non-communicable diseases, no difference was detected for hypertension prevalence. Within communicable diseases, there was no difference for rabies suspected bites but acute haemorrhagic gastroenteritis significantly decreased. The infant mortality rate and under five-year child mortality rate significantly increased during the FP period.

**Conclusion:** Primary care services should be reorganized and integrated with public health services.

KEY MESSAGES

- During the health reforms period, curative health services e.g. outpatient healthcare services were better, but preventive health services, e.g. surveillance of communicable diseases and follow-up of women and children were worse, except vaccinations.
- Preventive services should be reorganized and integrated with the primary care approach.

## Introduction

Turkish health reforms, known as the Health Transformation Programme, began in 2003 and brought some major changes in primary care services in Turkey. The most important change was the transition from a health centre (HC) system to the family physician (FP) system [1–3]. Before these reforms, the most significant development in primary care organization was the HC system, which began in 1961. The HC system depended on primary care services in a well-defined geographical region with a broad team containing several physicians, nurses, midwives and technicians, relying on home visits. [4] People living in the same region received healthcare services from the same doctor. In this system, patients could not select their doctor. This team-based system depended on a multi-sectoral healthcare service integrating both preventive and curative services [3,4]. The HC period, also called ‘the socialization of health services’, aimed to bring integrated primary care and public health services to even the remotest rural area [5].

FP services started in 2004, and they were widely distributed across Turkey by the end of 2010. The main difference between HC and FP systems is that the latter involves individual family practices. Everyone can select his/her family physician. Geographical borders, which were defined in the HC system, have been removed so that FPs can provide health services to people from all regions. In the FP system, the number of team members is reduced and now the team is composed of a single doctor and only one nurse or midwife. In the new system, an organization called a Community Health Centre is also established in order to provide public health services which are not supplied by FPs, such as school vaccinations, environmental health services, surveillance of communicable diseases, cancer screening programmes and health promotion activities to address high smoking rates and health-risk factors such as obesity [3].

Turkish primary care reforms also have a meaningful similarity with the reforms in former socialist European countries during the 1990s. These reforms were mainly funded by the World Bank and supported by the WHO [6]. The Turkish Ministry of Health support the health reforms but some other academics criticize them. According to the supporters, the primary objective of reforms is to improve the efficiency, governance, universal coverage and patient satisfaction [1,3]. However, opponents criticize privatization of healthcare services, inequalities in health and the new performance payment system, which led to increased patient examinations, operations and health shopping by patients [7,8]. Also, they argue that FPs had a heavy workload, stress and exhaustion after the reforms in Turkey [9].

It is important to investigate the outcomes of primary care reforms because these changes affect all general practitioners (GPs). This study aims to compare health status indicators in the HC period (between 2003 and 2007) with the FP period (between 2008 and 2012) in Manisa province, Turkey.

## Methods

### Study area

Manisa is in the western part of Turkey with a population of 1.3 million in 2012. People over 65 years old formed 8.0% and 9.6% of the population in years 2003 and 2012, respectively. While 73.9% of the population was living in urban areas, this figure decreased to 67.2% in 2012. Per 2012 statistics, the population of Turkey is 75.6 million.

### Study design

The study utilized time series analysis, using data that were collected at regular intervals. In Manisa district, the transition to FP in primary care services took place in 2008. Therefore, the data related to HC period between 2003 and 2007 and the data related to FP period between 2008 and 2012 were analysed for each five-year period. The data relating to the HC period was obtained from statistical yearbooks of Manisa Provincial Health Directorate and from the Primary Health Care Statistics, whereas the data associated with the FP period was obtained from the family physician information system. All data were also obtained electronically, by month. However, some data relating to maternal deaths, laboratories, family planning and distribution of causes of death could only be obtained on an annual basis. As 2011 and 2012 data related to family planning services were not available, they were not taken into consideration in this paper.

The dependent variables of the study are vaccination rates (%), follow-up programmes for infants, children, women and pregnant women (mean follow-up visit per person, per year), prevalence of communicable and non-communicable diseases (per ten thousand) and death rates (per thousand). The main elements of primary care reforms (selected variables, outcomes, measures, and conceptual framework of the study) are summarized in Table 1.

**Table 1.** Main elements of primary care reforms, outcomes and conceptual framework of the study.

**Main elements ofHC period(before primary care reforms)**	**Main elements ofFP period(after primary care reforms)**	**Outcomes or variableslinked toprimary care reforms**	Measures	**Conceptual framework(themes of the study)**
Broad team with 8–10 members (mainly home visits and follow-ups)	Narrow team with two members (mainly examination in the family physician office, limited home visits)	Follow-ups - Infant (0–11 months) - Children (1–4 years) - Pregnant women - Women (15–49 years) Vaccinations - Measles - BCG - HBV III - DPT III Mortality rates - Infant death rate - Postneonatal infant death rate - Under five years mortality rate - Crude death rate	Mean (per infant/per year) Mean (per child/per four years) Mean (per pregnant women) Mean (per women/per year) per cent per cent per cent per cent per thousand per thousand per thousand per thousand	Preventive health services
No selection of FP	Free selection of FP	Average number of outpatient services Non-communicable diseases - Hypertension	Mean (per person/per year) Prevalence (per ten thousand)	Healthcare utilization
Integrated health services (HCs for primary care and public health services)	Very weak or no integration (Family HCs for primary care; community HCs for public health services)	Communicable diseases - Rabies suspected animal bites - Acute haemorrhagic gastroenteritis	Incidence (per ten thousand) Incidence (per ten thousand)	Integration of health services
No extra payment or penalty	Pay for performance system (negative performance system for infant follow-ups, pregnant women follow-ups and infant vaccinations, but no penalty for women and children follow-ups) Extra payment for people who registered on FP’s list (per capita payment)	Follow-ups - Infant (0–11 months) - Children (1–4 years) - Pregnant women - Women (15–49 years) Vaccinations - Measles - BCG - HBV III - DPT III Average number of outpatient services	Limits Six times, per infant/per year Four times, per child/per four years Four times, per pregnant women Twice per woman/per year 90% 90% 90% 90% Mean (per person/per year)	Performance system

HC: health centre; FP: family physician; BCG: Bacillus Calmette–Guérin vaccine; HBV III: hepatitis B vaccine third dose; DPT III: diphtheria, pertussis (whooping cough), and tetanus vaccines third dose.

Follow-ups and vaccinations were chosen as dependent variables due to a strong link with primary care services. Hypertension and rabies-suspected bites were selected as being the most common non-communicable and communicable diseases. Measles and BCG vaccinations were selected due to the high incidence of measles and tuberculosis. DPT III and hepatitis B III vaccinations were chosen because infants are vaccinated at least three times in the first six months with these vaccines. Acute haemorrhagic gastroenteritis was selected for the following reasons: strong link to public health services, environmental health, food–water hygiene and sanitation. Death rates were selected as they are affected by primary care services and public health services. An average number of outpatient services for acute/chronic diseases were selected to detect the effect of patients’ freedom to choose GP rather being assigned a GP.

### Statistical analysis

We used segmented linear regression, which divides a time series into pre- and post-intervention segments. We chose January 2008 as the intersection point between segments (2003–2007 and 2008–2012) when the regulation for family healthcare system was introduced in Manisa. A linear regression model has two parameters: the level and slope. Therefore, the difference between the two segments of regression lines can be quantified by testing the change in these two parameters. A change in level between the pre- and post-intervention segments indicates a step-change, and a change in slope indicates a change in trend (Equation 1). Y_t_ is the aggregated outcome variable measured at each equally-spaced time point t. In this study, we used monthly data. β_1_ represents the coefficient of slope prior to intervention. β_2_ represents change in level in the period immediately following intervention initiation (compared to the counterfactual), β_3_ represents the difference between of pre- and post-intervention trends [10,11].

(1) $$Y_{t}=\beta_{0}+\beta_{1}T_{t}+\beta_{2}X_{t}+\beta_{3}X_{t}T_{t}+e_{t}$$

The analyses were conducted with STATA 11.0 and the values were presented with their 95% confidence intervals and statistical significance was considered for *P*-value <.05.

Permission was obtained from Manisa Provincial Health Directorate. Ethical Committee approval was given by Dokuz Eylul University Ethical Committee for non-invasive clinical research (study number 2012/11–14).

## Results

### Follow-up programmes

**Figure 1.**
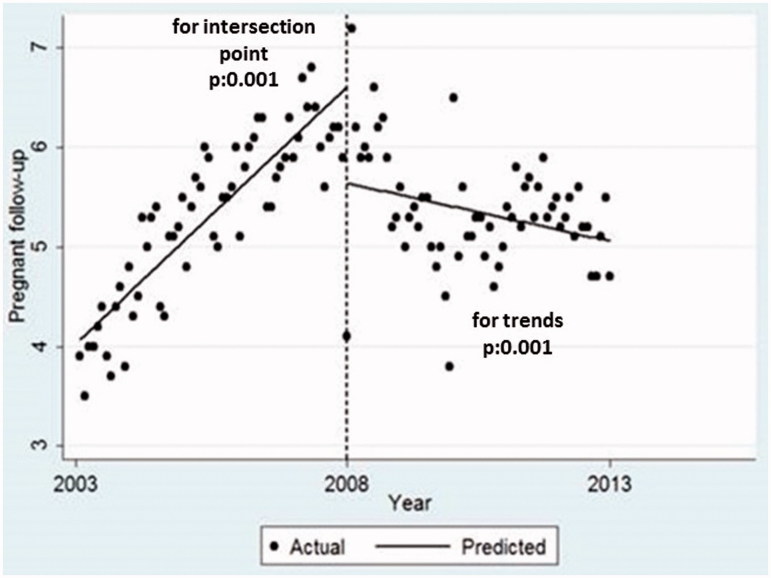
Pregnant women mean follow-up visits (per person/per year), intersection points and trends comparing health centre and family physician periods.

The predicted mean frequency of follow-up visits at the end of the HC period in infants was 10.5 per year, in pregnant women, 6.6 and women in general, 1.8. These were significantly higher than the mean frequency of follow-up visits predicted for the first year of FP period in infants: 6.8, pregnant women: 5.6 and women in general: 1.0. Following the investigation of the follow-up programmes, we observed a significant decrease in all intersection points and follow-up visits during the transition from HC period to the FP period. Furthermore, during the first five years of the FP period, while the number of follow-up visits of infants had increased to the previous level, the trends for pregnant women follow-up were significantly lower and opposite compared to HC period (Table 2, Figure 1).

**Table 2.** Follow-up visits, trends and differences comparing health centre and family physician periods.

	Mean follow-up visits (per people/per year)				Trends			
Indicators	HC period (95%CI)	FP period (95% CI)	Difference	*P*	HC period (95% CI)	FP period (95% CI)	Difference	*P*
Infant (0−11 months)	10.5 (10.0−11.02)	6.76 (5.88−7.64)	−3.83 (−2.83−4.82)	.001	0.06 (0.04−0.07)	0.03 (0.01−0.05)	−0.03 (−0.06, −0.01)	.001
Children (1−4 years)	2.11 (1.71−2.50)	1.16 (0.41−1.91)	−0.95 (−0.91−1.80)	.030	0.002 (−0.008, 0.013)	0.03 (−0.02,0.03)	0.003 (−0.023,0.029)	.812
Pregnant women	6.60 (6.31−6.82)	5.64 (5.14−6.14)	−0.97 (−1.56−0.38)	.001	0.04 (0.03−0.05)	−0.01 (−0.02, 0.001)	−0.05 (−0.07, −0,04)	.001
Women (15−49 years)	1.76 (1.29−2.21)	0.96 (0.81−1.20)	−0.81 (−1.35−0.26)	.004	0.005 (−0.01,0.02)	−0.002 (−0.01−0.004)	−0.01 (−0.02,0.01)	.321

### Vaccinations

There was a significant step increase in vaccination rates for BCG and measles during the transition period (Table 3). We observed a downward trend for BCG vaccination, which was significantly different. Hepatitis B III vaccination rates increased but the difference was not significant. However, the hepatitis B III vaccination rates tended to decrease later on in the FP period. Step change in DPT III vaccination rate was not significant and trends did not differ significantly in both periods.

**Table 3.** Vaccination rates, trends and differences comparing health centre and family physician periods.

	Vaccinations (%)				Trends			
Indicators	HC period (95% CI)	FP period (95% CI)	Difference	*P*	HC period (95% CI)	FP period (95% CI)	Difference	*P*
BCG (Percent)	101.95 (95.47-108.44)	115.28 (105.82-124.74)	13.29 (0.66, 25.92)	.039	0.04 (−0.17−0.25)	−0.27 (−0.54−0.007)	−0.31 (−0.63, −0.01)	.042
Measles (%)	86.42 (71.19−101.64)	118.46 (104.77−132.16)	32.27 (11.68, 52.84)	.002	−0.23 (−0.66, 0.21)	−0.38 (−0.75−0.002)	−0.16 (−0.73,0.41)	.580
HBV III (%)	103.80 (92.38−115.23)	117.14 (108.89−125.39)	13.23 (−1.36, 27.82)	.075	0.11 (−0.17, 0.39)	−0.30 (−0.52, −0.09)	−0.41 (−0.76, −0,06)	.021
DPT III (%)	100.51 (95.31−105.70)	100.09 (82.68−117.50)	−0.43 (−18.65, 17.80)	.963	0.089 (−0.41, 0.59)	0.096 (−0.34, 0.53)	−0.089 (−0.41, 0.59)	.725

### Healthcare utilization

**Figure 2.**
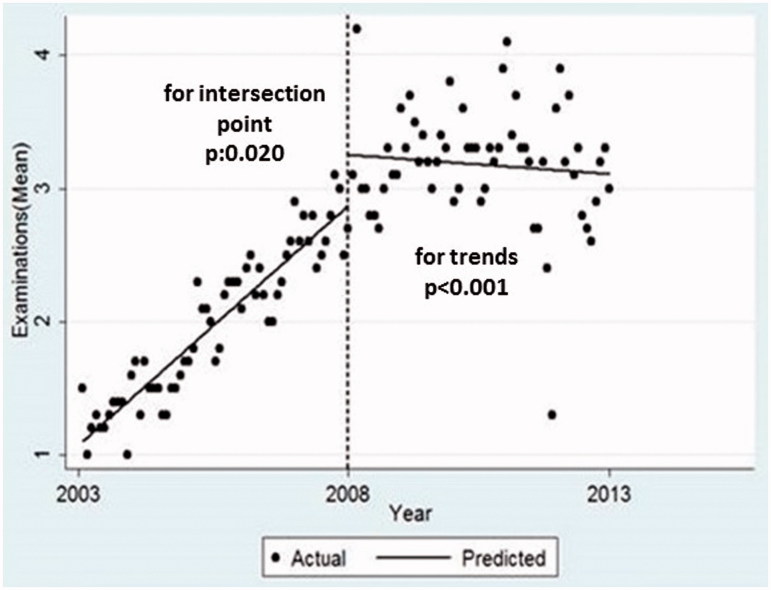
Average number of outpatient services (per person/per year), intersection points and trends comparing health centre and family physician periods.

At the beginning of the FP period, the number of patients who had undergone physical examination has increased. The predicted mean number of physical examinations per person at the start of the FP period 3.3 was significantly higher than the HC period 2.8. However, this showed a downward trend over the years and the difference between the slopes was significant (Figure 2).

Among communicable diseases, acute gastroenteritis significantly decreased at the beginning of the FP period, whereas suspected rabies bites remained the same (Table 4). Both diseases had a significant declining trend during the FP period compared to the HC period. Among non-communicable diseases, hypertension showed an increasing trend in both periods, however, the trend during the FP period was lower compared to the HC period.

**Table 4.** Morbidity rates, trends and differences comparing health centre and family physician periods.

	Average number of outpatient services and disease rates				Trends			
Indicators	HC period (95% CI)	FP period (95% CI)	Difference	*P*	HC period (95% CI)	FP period (95% CI)	Difference	*P*
Average number of outpatient services (Mean, per person, per year)	2.84 (2.69, 2.99)	3.25 (2.97, 3.52)	0.379 (0.059, 0.697)	.020	0.029 (0.025, 0.034)	–0.024 (0.012, 0.007)	–0.032 (–0.042, –0.022)	<.001
Hypertension (per ten thousand)	31.29 (28.83, 33.74)	26.05 (18.33, 33.78)	–6.34 (–14.87, 2.21)	.144	1.10 (0.940, 1.271)	0.416 (0.157, 0.674)	–0.69 (–0.99, –0.39	.001
Rabies suspected animal bites (per ten thousand)	31.89 (26.77, 37.01)	36.94 (26.12, 47.77)	4.53 (–6.63, 15.69)	.422	0.519 (0.279, 0.759)	–0.171 (–0.486, 0.145)	–0.69 (–1.11, –0.264)	.002
Acute haemorrhagic gastroenteritis (per ten thousand)	3.98 (3.13, 4.83)	1.79 (1.45, 2.13)	–2.24 (–2.99, –1.49)	<.001	0.052 (0.033, 0.0719)	–0.011 (–0.020, –0.002)	–0.063 (–0.09, –0.04)	<.001

### Mortality

The infant mortality rate was 9.2 per thousand, the postneonatal infant mortality rate was 3.3 per thousand, and the under-five years mortality rate was 11.1 per thousand, in the HC period (Table 5). The predicted rates showed a significant increase in the FP period for the infant and child mortality rates. However, these rates showed a downward trend over the years, approaching the rates prevalent in the HC period. Crude death rate showed an increasing trend in both periods but did not differ significantly between periods and the segmentation point showed no significant difference.

**Table 5.** Mortality rates, trends and differences comparing health centre and family physician periods.

	Mortality rates				Trends			
Indicators	HC period (95% CI)	FP period (95% CI)	Difference	*P*	HC period (95% CI)	FP period (95% CI)	Difference	*P*
Infant mortality rate (per thousand)	9.21 (7.83−10.60)	14.51 (12.81−16.20)	5.37 (3.13−7.61)	<.001	−0.08 (−0.13, −0.38)	−0.119 (−0.16, −0.074)	−0.04 (−0.01, 0.03)	.261
Postneonatal infant mortality rate (per thousand)	3.32 (2.20−4.44)	5.02 (3.92−6.21)	1.78 (0.16,3.41)	.032	−0.042 (−0.08, −0.01)	−0.0359 (−0.066, −0.0053)	0.01 (−0.41, 0.05)	.812
Under 5 years mortality rate (per thousand)	11.09 (9.61−12.57)	15.06 (13.85−17.34)	4.60 (2.25−6.94)	.001	−0.09 (−0.14, −0.04)	−0.131 (−0.176, −0.084)	−0.04 (−0.11, 0.03)	.256
Crude death rate (per thousand)	3.71 (2.72−4.69)	2.05 (0.54−3.55)	−1.67 (−3.47, 0.13)	.069	0.01 (−0.27, 0.04)	0.06 (0.02−0.10)	0.054 (−0.003, 0.111)	.065

## Discussion

### Main findings

In this study, the first important finding was the decreased number of follow-up visits for high-risk groups in the year 2008, which was the transition time for health reforms in Manisa province. This finding could be due to the change in follow-up charts during the FP period and the decrease in the required number of follow-up visits per year from nine to six in infants, from 12 to four in pregnant women and from eight to four in children [12]. Another reason could be the reorganization of health services, which health managers mentioned as an important issue. According to the health managers, the system changed very rapidly and over a short time [13]. Thus, the significant decrease in mean values of follow-ups at the beginning of the FP period was predictable. However, trends in follow-ups vary in the FP period: while the number of follow-up visits for infants shows an increasing trend, the follow-up visits of pregnant women show a decreasing trend. The upward trend for infants could be related to the frequency of compulsory vaccination appointments compared to the number of required appointments for pregnant women. Reason for the downward trend for pregnant women’s visits could reflect a preference to see their obstetrics and gynaecology specialists rather than their GP, especially when we consider that 95% of births take place in hospitals.

Although there were no problems associated with the vaccination rates before and after the primary care reforms, in the FP period vaccination rates were usually over 100%. This is likely due to the high number of unregistered children in the region. According to health managers, there were approximately 10% unregistered children in the HC period, and records were not held electronically [13]. After the primary care reforms, all children’s records were corrected and every child was registered electronically with the nearest FP. This created rates of vaccination which were over 100%.

Our study also showed that the infant mortality rates increased after the transition to the FP period. This increase could be due to improvements in recording practices, especially for infants. Issues related to death records, such as lack of data in the HC period, have been mentioned in other studies [13–15]. Conversely, there was no statistical difference for the trends in infant mortality rates, even though we observed a decrease in the FP period. Thus, the increase in infant mortality rates is not seen as a negative consequence of health reforms.

### Performance system and health reforms

Most previous studies demonstrated that follow-ups and vaccinations related to the new performance-based payment system, and usually increased during the FP period [9,16–18]. Per the performance-based payment system in Turkey, a minimum number of monthly follow-up visits were required for infants and pregnant women and this system was started in 2008–2009 in that region. The significantly increasing trends for follow-up of infants, and vaccination rates are likely to be related to this payment system. However, the decline in number of follow-up visits for women and children should be taken as a warning in our study. FPs tend to perform fewer follow-up visits for these groups, which are not included in the performance-based payment system. Another problem may also be encountered in family planning services, which is likely to follow the same pattern as the follow-up of women in the FP period.

### Healthcare utilization

The mean number of outpatient services during the FP period is significantly higher when compared with the HC period. This increase can be related to consumer and market-oriented policies implemented by the primary care reforms. However, there is a plateau after the FP approach was implemented. This situation could be related to the end of some funding arrangements (there was a unique private financial fund in some HCs) in the FP approach.

According to some studies, the effect of the health reforms on primary care services in Turkey resulted in better quality of healthcare services and increased patient satisfaction [3,19]. However, other studies contradict this finding. Specifically, they criticize the strength and quality of primary healthcare services in Turkey and the working conditions of FPs [20,21]. When we look at the implications of health reforms in other countries in the region, we see almost same results. In many healthcare systems in Europe, GP/FPs are not only healthcare providers but also small-scale entrepreneurs. In most of central and eastern European countries, there is an inclination towards independent practitioner status for GPs [22]. The aim of Turkish primary care reforms are also change the stature of GP/FPs as self-employed physicians in the future.

Among non-communicable diseases, no difference was detected for hypertension, but the trend is significantly decreased. However, this apparent decrease may be attributable to lack of reliable records and referral system. Turkey is one of the few countries without a national system of gate-keeping. Lack of a referral system resulted in increased specialist visits in Turkey after the initiation of the health transformation programme, and therefore, non-communicable diseases’ records are not reliable at the first level [23].

Rabies suspected bites and acute gastroenteritis significantly decreased from 2008 to 2012 in the region. These data are consistent with Turkish Ministry of Health and Turkish Statistical Institute data [23,24]. This finding could be related to improvements made in the infrastructure (such as water supply and waste collection systems), and healthy environment projects organized by municipalities. Moreover, the definition of acute gastroenteritis has been changed to acute ‘haemorrhagic’ gastroenteritis in the FP period, which could be another reason for the decreasing gastroenteritis trend. However, failure to investigate contacts and source of infection and surveillance of communicable diseases are still important problems in the FP period, according to health managers [13]. Since there are no data on this issue in Manisa records after 2009, the surveillance reports could not be evaluated. These findings indicate that consistent with other studies, there was not enough control of infectious diseases and environmental health services by the Ministry of Health [13,25,26]. These surveillance problems show an integration problem between primary care and public health services. However, there are some contradictory findings for other candidate countries of EU, which have similar integration problems while also having well-established surveillance systems [27].

### Policy recommendations

Follow-up services should be empowered especially for women and children and the quality of these follow-ups should also be evaluated. Mortality–morbidity and risk factors data should be in the national health information system and established national registries. A surveillance system should be properly maintained. Reliable records for non-communicable diseases and surveillance of communicable diseases should be supplied with a coordination of primary care and public health services [28]. Finally, primary care services should be integrated into public health services. The Turkish Ministry of Health should intervene in coordination problems among the newly established divisions for primary care and public health services.

### Strengths and limitations

One of the strengths of this study is the use of segmented regression analysis to evaluate the effects of changes in primary care services provision introduced at a specific point in time on outcomes. The main limitation of the current study is insufficient data in some services such as family planning, communicable and non-communicable diseases. Furthermore, Manisa is a western city and therefore this limitation should also be considered. The eastern region of Turkey is poorer as economically and socially than the western part of country. Another limitation relates to comparing high-risk groups over a 10-year period. Our population may be getting older, more obese and more comorbid over time, and morbidity and the mortality rates could be affected by time. Similarly, there is another time effect: the first year of the FP period should be considered as a transitional period. This study had some methodological limitations. These include potential time-varying confounders, which were not considered and could affect the outcome. Seasonality and autocorrelation could be a problem in times series analysis which might bias the findings; however, we have taken this into account in autoregressive models. Another limitation is that some observations might follow a non-linear distribution, so using linear regression models might have caused over/underestimation of the expected values.

## Conclusion

Primary care services should be integrated into other services. There is a serious integration problem for these services in Turkey, for example, the lack of a referral system. Given this, we recommend that Community Health Centres be reorganized, especially for communicable diseases and environmental health services, to integrate them with the FP approach.
